# Dentoalveolar Transverse Changes in Adolescents Treated with Fixed Appliances or Clear Aligners: A Retrospective Comparative Study

**DOI:** 10.3390/children13101306

**Published:** 2026-09-25

**Authors:** Ömer Faruk Sari, Muhammed Hilmi Buyukcavus

**Affiliations:** 1 Department of Orthodontics, Faculty of Dentistry, Ankara Medipol University, 06100 Ankara, Turkey; 2 Private Practice, Buyukcavus Dental Clinic, 03100 Afyonkarahisar, Turkey; mhbuyukcvs@gmail.com

**Keywords:** adolescents, dentoalveolar expansion, transverse arch development, clear aligners, fixed orthodontic appliances, digital dental models

## Abstract

**Highlights:**

**What are the main findings?**

- Measurable dentoalveolar transverse increases were observed in all four treatment groups, although the magnitude and distribution of these changes differed among groups.
- Greater transverse increases at several maxillary levels were observed in the Damon Q group, whereas comparatively smaller changes in maxillary first-molar crown inclination were observed in the ClearCorrect group. These findings represent between-group patterns observed during treatment and should not be interpreted as isolated appliance effects.

**What is the implication of the main finding?**

- Fixed appliances and clear aligners may produce distinct patterns of transverse arch development, which should be considered when selecting treatment mechanics for adolescent patients.
- Appliance-related differences should be interpreted cautiously because physiological growth and other treatment-related factors may also contribute to the observed changes.

**Abstract:**

**Background:** Dentoalveolar transverse constriction is frequently encountered during orthodontic treatment in adolescents. Although both fixed appliances and clear aligners can produce transverse dental arch changes, comparative evidence regarding their expansion patterns in growing patients remains limited. This study aimed to compare dentoalveolar transverse changes associated with two fixed treatment groups and two clear aligner systems in an adolescent population. **Methods:** This retrospective, non-randomized comparative study included 85 adolescents treated without extractions using conventional MBT fixed appliances (*n* = 22), Damon Q passive self-ligating appliances (*n* = 19), Invisalign (*n* = 21), or ClearCorrect (*n* = 23). Pretreatment (T0) and post-treatment (T1) digital dental models obtained using a 3Shape TRIOS intraoral scanner were evaluated for maxillary and mandibular transverse arch dimensions, projected palatal area, and maxillary first-molar crown inclination. Intergroup differences in treatment-related changes were compared among the four treatment groups. **Results:** Significant increases in maxillary transverse dimensions were observed after treatment in all four groups. The greatest mean increases at several maxillary levels were observed in the Damon Q group, including intercanine width (3.66 ± 1.34 mm) and first-molar width (3.69 ± 1.70 mm). In the ClearCorrect group, the mean increases were 3.12 ± 1.90 mm for intercanine width and 2.46 ± 1.42 mm for first-molar width. Projected palatal area increased in all groups, with the greatest mean increase observed with Damon Q (115.16 ± 29.70 mm^2^). Exploratory assessment of maxillary first-molar clinical crown inclination showed comparatively smaller changes in the ClearCorrect group than in the other treatment groups. **Conclusions:** All four treatment groups demonstrated increases in dentoalveolar transverse dimensions in this adolescent sample, although the magnitude and pattern of the observed changes differed among groups. Greater transverse increases at several maxillary levels were observed in the Damon Q group, whereas comparatively smaller changes in posterior crown inclination were observed in the ClearCorrect group. Because skeletal maturation was not directly assessed and an untreated control group was unavailable, these findings represent comparative changes observed during treatment and cannot distinguish appliance-related effects from physiological growth or other treatment-related factors.

## 1. Introduction

Transverse maxillary constriction is a clinically relevant component of malocclusion in children and adolescents and may be associated with posterior crossbite, dental crowding, and altered transverse occlusal relationships [1]. Accurate diagnosis requires differentiation between skeletal and dentoalveolar components because treatment strategies differ according to the underlying transverse discrepancy and the patient’s growth status [2,3]. Skeletal transverse deficiencies during growth may require orthopedic expansion, whereas mild-to-moderate dentoalveolar constriction may be managed through transverse dental arch development using fixed orthodontic appliances or clear aligners.

During adolescence, interpretation of treatment-related transverse changes is particularly relevant because orthodontic tooth movement occurs alongside ongoing craniofacial and dentoalveolar development. Fixed treatment groups can produce transverse arch changes through alterations in tooth position and crown inclination, with the magnitude and pattern of expansion potentially varying according to bracket and archwire characteristics [4]. Clear aligner therapy has also become increasingly used in younger patients and can produce clinically measurable transverse changes [5,6]. However, aligner-mediated expansion is predominantly dentoalveolar, and its predictability may vary among different regions of the dental arch [7,8]. Consequently, changes in arch width should be interpreted together with posterior tooth inclination rather than considering dimensional increases alone as evidence of controlled expansion.

Although transverse changes produced by fixed appliances and clear aligners have been investigated separately, direct comparisons of different treatment groups within a comparable adolescent population remain limited. Moreover, differences between conventional fixed appliances, passive self-ligating systems, and clear aligner systems may be reflected not only in the magnitude of transverse arch changes but also in their distribution across the dental arch and the accompanying changes in posterior crown inclination. Digital dental models provide a non-ionizing method for evaluating these dentoalveolar outcomes and are particularly suitable for repeated morphological assessment in younger populations [8,9,10].

However, each approach has inherent limitations when transverse correction is attempted. Conventional and self-ligating fixed appliances primarily achieve arch development through dentoalveolar tooth movement and changes in posterior crown inclination, and the resulting transverse response may be influenced by archwire form, bracket prescription, and the periodontal boundaries of tooth movement. Similarly, clear aligner expansion is predominantly dentoalveolar, may show reduced predictability in posterior regions, and is dependent on adequate patient compliance. Neither fixed appliances nor clear aligners should therefore be considered substitutes for orthopedic or surgically assisted expansion when a substantial skeletal transverse deficiency is present. These limitations highlight the importance of distinguishing dentoalveolar arch development from true skeletal expansion when comparing treatment approaches [8,9,10,11].

Therefore, the aim of this retrospective comparative study was to evaluate dentoalveolar transverse changes in adolescents treated without extractions using four treatment groups: conventional MBT fixed appliances, Damon Q passive self-ligating appliances, Invisalign clear aligners, and ClearCorrect clear aligners. Maxillary and mandibular transverse arch dimensions, projected palatal area, and maxillary first-molar crown inclination were compared between pretreatment and post-treatment digital dental models [9,10,11]. The null hypothesis was that there would be no significant differences among the four treatment groups in the magnitude or pattern of dentoalveolar transverse changes.

## 2. Materials and Methods

### 2.1. Study Design and Participants

This retrospective, non-randomized comparative study was conducted using the clinical records and digital dental models of patients who had undergone comprehensive orthodontic treatment at the our dental clinic. The study protocol was approved by the Ethics Commission of the Medical Faculty of Suleyman Demirel University, Isparta, Türkiye (approval no. 40; 26 January 2022) and was conducted in accordance with the principles of the Declaration of Helsinki. Written informed consent for orthodontic treatment and the use of clinical records for research purposes was obtained from all participants and, where applicable, from their parents or legal guardians.

The records of 106 consecutively screened patients were assessed for eligibility. Patients were eligible if they were adolescents in the late mixed or permanent dentition, presented with mild-to-moderate dentoalveolar transverse maxillary constriction, underwent non-extraction comprehensive orthodontic treatment with one of the four investigated treatment groups, and had adequate pretreatment (T0) and post-treatment (T1) digital dental models available for analysis. Dentoalveolar transverse constriction was identified from pretreatment clinical records and digital dental models based on reduced maxillary transverse dimensions, with or without posterior crossbite, in the absence of a skeletal transverse discrepancy requiring orthopedic maxillary expansion.

Patients were excluded if they had a craniofacial anomaly or syndrome, a history of previous orthodontic treatment, a skeletal transverse maxillary deficiency requiring rapid or other orthopedic maxillary expansion, tooth extraction as part of the comprehensive orthodontic treatment, incomplete clinical or digital records, or digital models of insufficient quality for standardized measurements. Patients treated with auxiliary expansion appliances were also excluded.

Because of the retrospective design, skeletal maturation indicators, including cervical vertebral maturation stages, were not consistently available and could therefore not be used as eligibility or stratification criteria. Accordingly, chronological age and pretreatment transverse measurements were evaluated to characterize baseline comparability among the treatment groups. The potential contribution of physiological growth to transverse changes was considered when interpreting the findings.

Treatment modality was determined during routine clinical care and was not assigned for research purposes. Selection of conventional MBT fixed appliances, Damon Q passive self-ligating appliances, Invisalign (Align Technology, Tempe, AZ, USA) clear aligners, or ClearCorrect (ClearCorrect LLC, Round Rock, TX, USA) clear aligners was based on clinical indication, patient preference, and appliance availability at the time of treatment. The American Board of Orthodontics (ABO) Discrepancy Index was obtained from the pretreatment records to characterize overall case complexity; it was not used as a specific measure of transverse discrepancy. Because treatment allocation was not randomized, other baseline characteristics that could potentially influence treatment response, including the amount of initial crowding, arch-length discrepancy, buccal segment relationships, and patient-related factors influencing compliance, were not systematically balanced or separately matched among the four groups. These variables were not consistently available in a standardized quantitative format in the retrospective records and were therefore not included as covariates in the present analysis. Consequently, residual confounding related to unmeasured baseline differences cannot be excluded.

**Figure 1:**
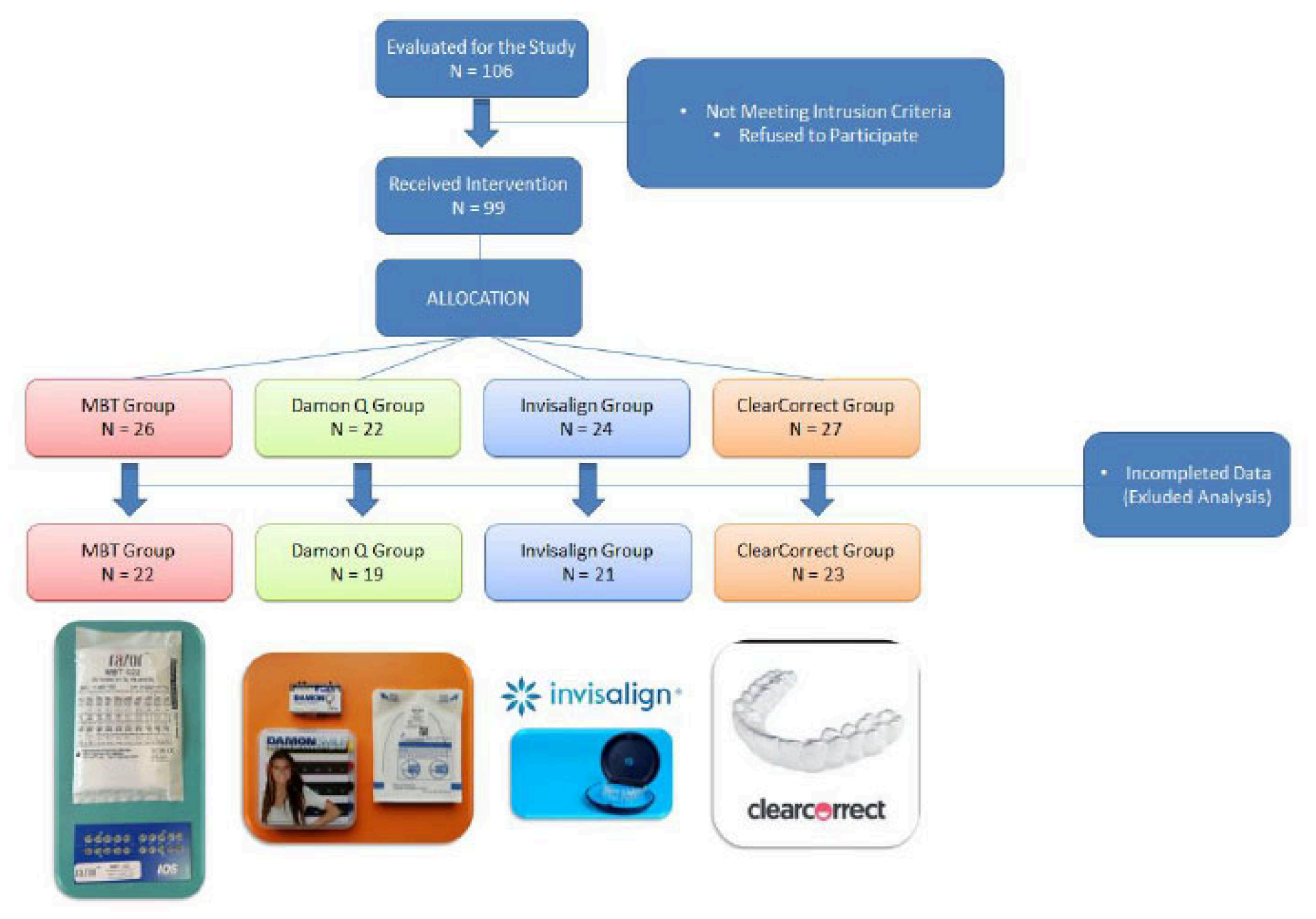
Flow diagram of patient screening, eligibility assessment, exclusions, and final allocation to the four treatment groups.

After application of the eligibility criteria, 99 patients remained. Fourteen patients were subsequently excluded because of incomplete data or records that were inadequate for the planned analyses, resulting in a final sample of 85 patients. The final study population comprised 22 patients treated with conventional MBT fixed appliances, 19 with Damon Q passive self-ligating appliances, 21 with Invisalign clear aligners, and 23 with ClearCorrect clear aligners (Figure 1).

### 2.2. Treatment Protocols

All patients underwent comprehensive non-extraction orthodontic treatment using one of the four investigated treatment approaches. All treatments were performed by the same orthodontist. Treatment procedures were applied consistently within each appliance group according to the corresponding clinical protocol; however, complete standardization across the four groups was not possible because the appliance systems inherently differed in archwire sequences, aligner staging, attachment use, and refinement requirements. No auxiliary maxillary expansion appliances or adjunctive mechanics specifically intended to increase transverse arch width were used during treatment.

#### 2.2.1. Conventional Fixed Appliance Group

Patients in the conventional fixed appliance group were treated with MBT-prescription stainless-steel brackets (Razor SS brackets, International Orthodontic Services [IOS], Stafford, TX, USA) with a 0.022-inch slot in our clinic. Alignment and leveling were performed using a sequential archwire protocol consisting of 0.014-, 0.016-, and 0.018-inch nickel-titanium (NiTi) archwires, followed by 0.016 × 0.016-, 0.016 × 0.022-, 0.017 × 0.025-, and 0.019 × 0.025-inch NiTi archwires. A 0.019 × 0.025-inch stainless-steel archwire was used during the finishing phase. Pretreatment digital models were obtained before bracket placement, and post-treatment models were obtained after completion of active orthodontic treatment.

#### 2.2.2. Passive Self-Ligating Fixed Appliance Group

Patients in the passive self-ligating group were treated with Damon Q brackets (Ormco Corporation, Orange, CA, USA) with a 0.022 × 0.028-inch slot. Alignment and leveling were performed using sequential 0.014- and 0.016-inch copper nickel-titanium (CuNiTi) archwires, followed by 0.014 × 0.025-, 0.016 × 0.025-, and 0.018 × 0.025-inch CuNiTi archwires. A 0.019 × 0.025-inch stainless-steel archwire was used during the finishing phase. Pretreatment digital models were obtained before bracket placement, and post-treatment models were obtained after completion of active orthodontic treatment.

In both fixed appliance groups, preformed archwires were used throughout treatment. Archform selection was based on the pretreatment arch morphology and clinical treatment objectives. The archwire sequence and treatment principles were applied consistently within each appliance group.

#### 2.2.3. Invisalign Clear Aligner Group

Patients in the Invisalign group were treated with Invisalign clear aligners (Align Technology, San Jose, CA, USA). Digital impressions were obtained using a 3Shape intraoral scanner (3Shape A/S, Copenhagen, Denmark), and virtual treatment planning was performed before aligner fabrication. Transverse arch development was planned sequentially, generally beginning in the posterior region and progressing anteriorly to the premolars and canines. Expansion was programmed in increments of approximately 0.25 mm per stage. Patients were instructed to wear their aligners for at least 22 h/day and were monitored at regular follow-up visits.

#### 2.2.4. ClearCorrect Clear Aligner Group

Patients in the ClearCorrect group were treated with ClearCorrect aligners (ClearCorrect LLC, Straumann Group, Round Rock, TX, USA). Digital impressions were obtained using a 3Shape intraoral scanner, and treatment was planned using the ClearPilot platform. Transverse arch development was planned sequentially, generally beginning in the molar region and progressing anteriorly to the premolars and canines. Expansion was programmed in increments of approximately 0.25 mm per stage. Patients were instructed to wear their aligners for at least 22 h/day and were monitored at regular follow-up visits.

In both clear aligner groups, the number of treatment stages was individualized according to the clinical treatment plan. Refinement aligners were prescribed when clinically indicated and followed the same general transverse treatment principles as the initial aligner series. No auxiliary expansion appliances were used. Attachments were prescribed according to individual alignment and tooth-movement requirements; dedicated expansion attachments were not routinely used. Transverse overcorrection was not routinely incorporated into the initial virtual treatment plan.

Compliance with aligner wear was assessed during routine follow-up visits based on clinical evaluation and patient-reported wear time. Objective electronic wear-time monitoring was not available; therefore, compliance data should be interpreted as clinically assessed rather than objectively quantified.

### 2.3. Digital Model Analysis and Outcome Measurements

Pretreatment (T0) and post-treatment (T1) digital dental models were obtained using a 3Shape intraoral scanner (3Shape A/S, Copenhagen, Denmark) and exported in Standard Tessellation Language (STL) format. Before measurement, the maxillary T0 and T1 models were registered within a common coordinate system using stable palatal anatomical landmarks, primarily the medial palatal rugae region, to reduce differences related to model orientation. Linear and angular measurements were subsequently performed using the registered digital models.

**Figure 2:**
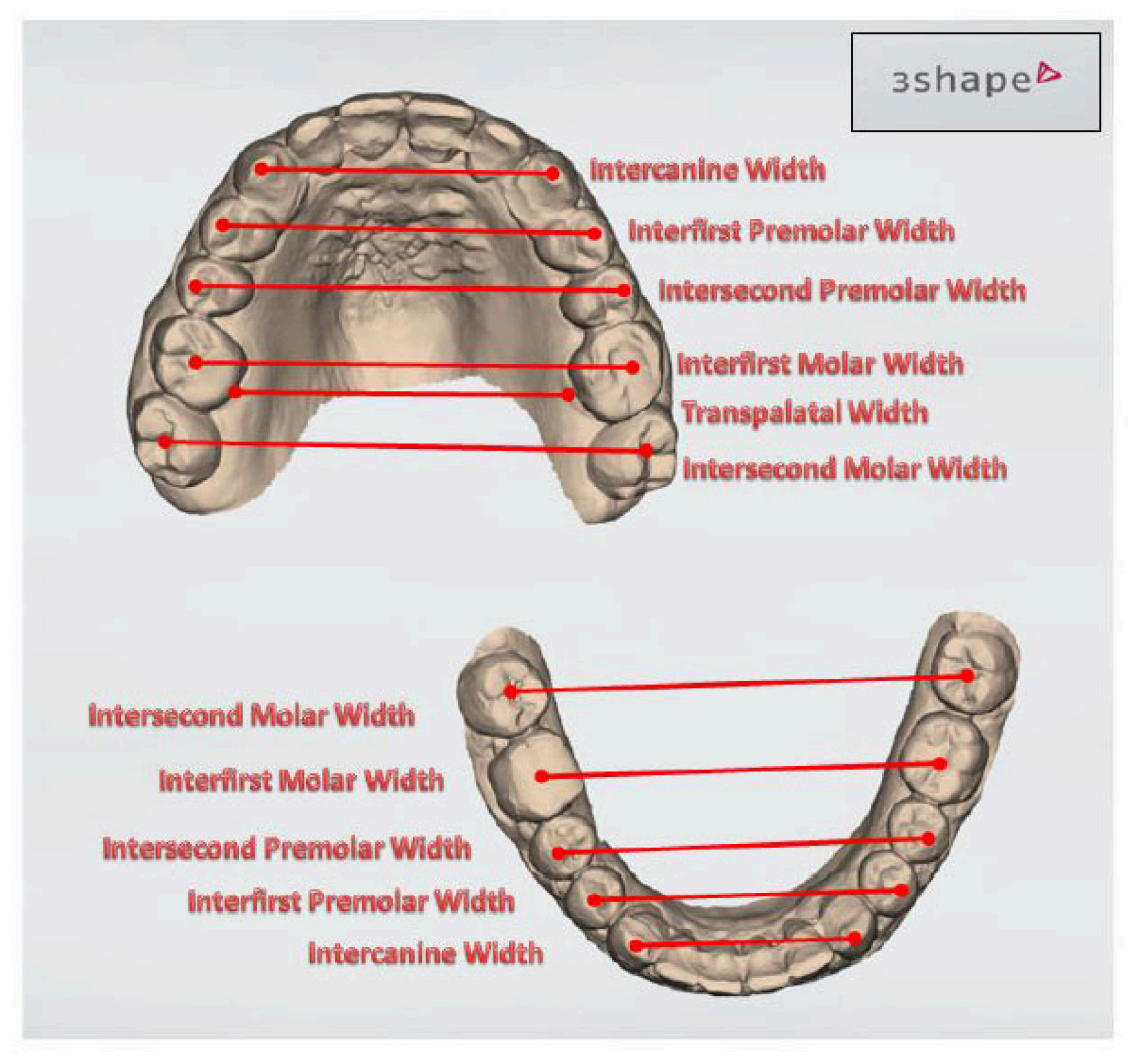
Digital model landmarks and reference points used for transverse dental arch measurements.

Transverse dental arch dimensions were assessed separately in the maxilla and mandible. Intercanine width was defined as the linear distance between the cusp tips of the right and left canines. Inter-first-premolar and inter-second-premolar widths were measured between the corresponding buccal cusp tips. Inter-first-molar and inter-second-molar widths were measured between the central fossae of the corresponding right and left molars. Maxillary transpalatal width was defined as the distance between the palatal cervical margins of the right and left first permanent molars. The landmarks and reference points used for these transverse dental arch measurements are illustrated in Figure 2.

**Figure 3:**
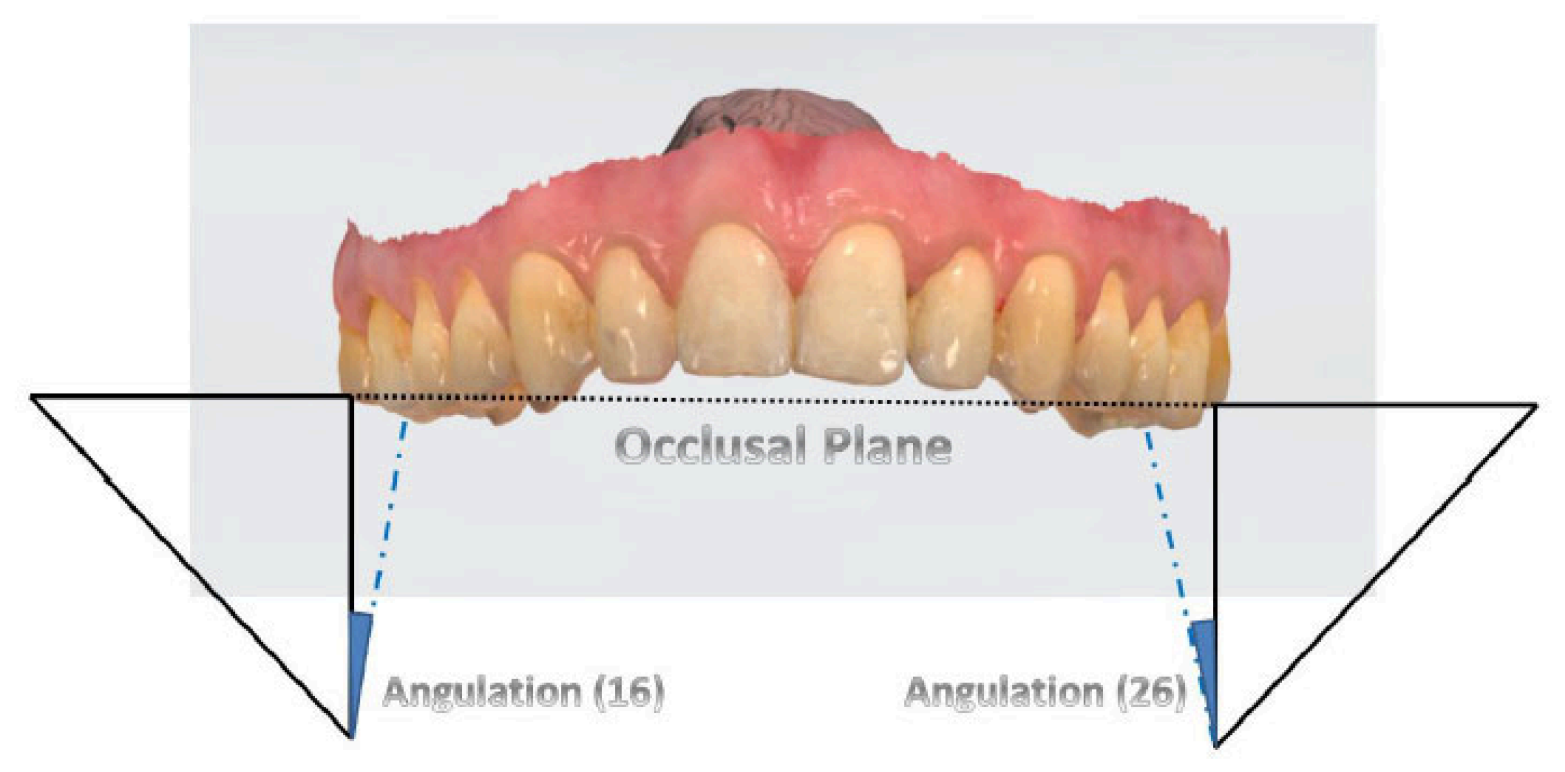
Digital-model method used to assess buccopalatal crown inclination of the maxillary right and left first permanent molars.

Buccopalatal crown inclination of the maxillary right and left first permanent molars (UR16 and UL26) was evaluated using standardized reference planes constructed on the registered digital models. An occlusal reference plane was established using the cusp tips of the molars, premolars, and canines. For each first molar, a paracoronal plane perpendicular to the occlusal reference plane was constructed. A curve following the long axis of the clinical crown was generated, and a best-fit line was determined using the most occlusal and gingival points of the crown profile. Molar crown inclination was defined as the angle between this best-fit crown axis and the corresponding paracoronal plane. The digital-model procedure used for the assessment of maxillary first-molar crown inclination is illustrated in Figure 3.

The angular measurements derived from the STL models represent clinical crown inclination rather than true three-dimensional tooth inclination or root torque. Because CBCT imaging was not available, root position, alveolar bone relationships, and true buccolingual torque could not be assessed. Accordingly, angular changes were interpreted exclusively as changes in crown inclination.

**Figure 4:**
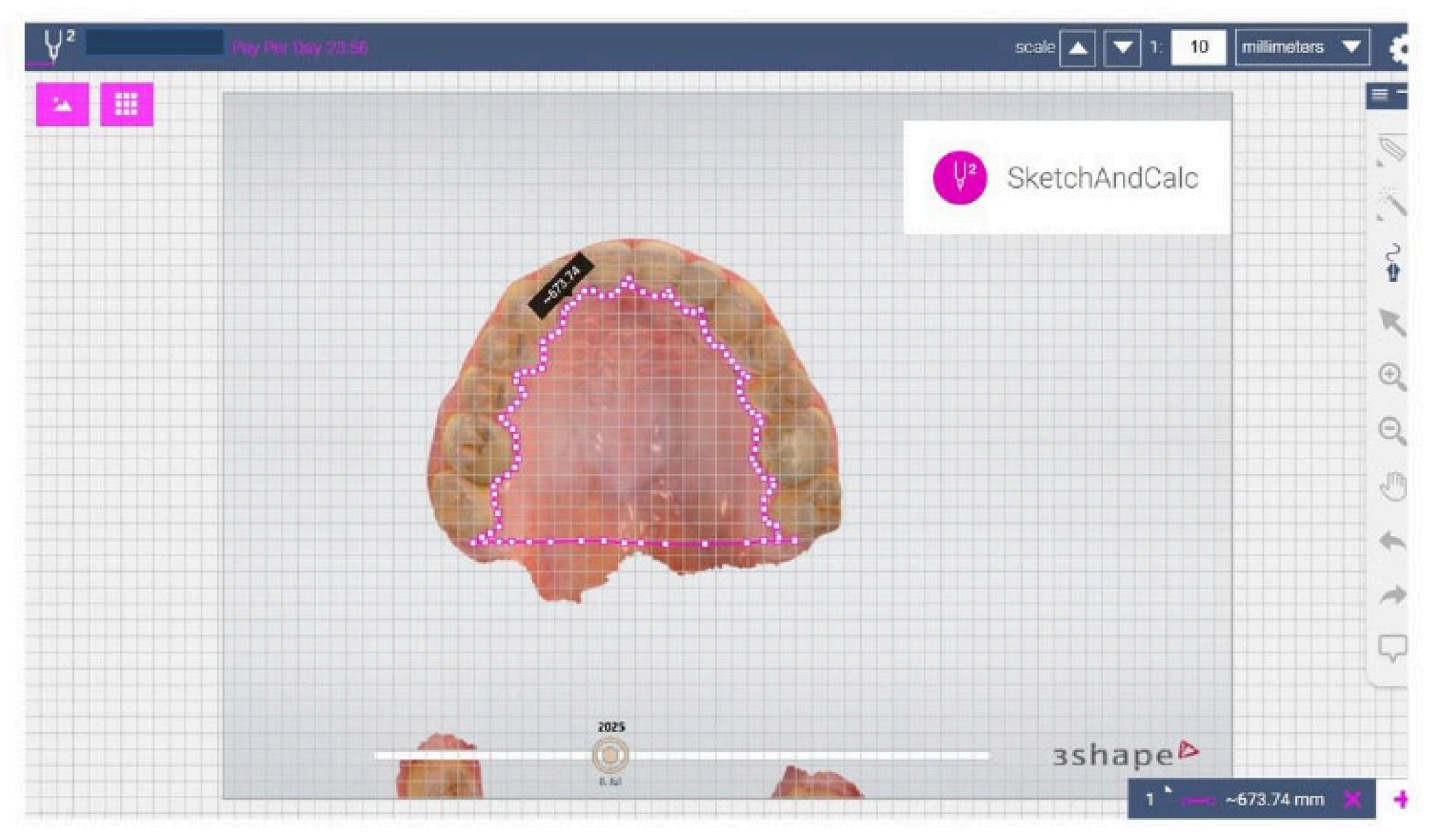
Standardized two-dimensional projection and boundary definition used for measurement of the projected palatal area.

Projected palatal area was assessed from the maxillary digital models using SketchAndCalc™ (Cadmium Design, Beaverton, OR, USA) software. Before area measurement, the registered T0 and T1 maxillary models were maintained within the same standardized coordinate system used for the other digital-model measurements. This registration was based primarily on stable medial palatal rugae landmarks and was used to minimize differences in model orientation between time points. The registered models were then projected onto the same two-dimensional reference orientation for area assessment. The palatal boundary was traced consistently along the cervical margins of the maxillary teeth and posteriorly to a line connecting the distal aspects of the right and left second permanent molars (Palatal A-line). The same anatomical boundary definition and tracing procedure were applied at T0 and T1 and across all treatment groups. The enclosed projected area was calculated in mm^2^. The standardized two-dimensional projection and boundary definition are illustrated in Figure 4. Because this measurement represents a two-dimensional projection of a three-dimensional curved palatal surface rather than the true three-dimensional surface area, it was defined as projected palatal area throughout the study and was interpreted as a complementary morphological indicator of transverse arch change.

All measurements were performed by a single calibrated examiner using the same measurement definitions and reference procedures at T0 and T1. Because appliance-related information could be identifiable from the clinical records and/or digital models, complete blinding of the examiner to treatment modality could not be ensured. To reduce measurement variability, standardized landmark definitions, model-registration procedures, and reference-plane constructions were applied consistently across all groups and time points.

### 2.4. Measurement Reliability

To assess intra-examiner reliability, measurements were repeated by the same calibrated examiner on 20 randomly selected digital models after a two-week interval. Random error was quantified using the Dahlberg formula, and intra-class correlation coefficients (ICCs) were calculated to assess agreement between repeated measurements. ICC values were interpreted according to established reliability criteria. For the reliability assessment, the examiner was blinded to the values obtained during the initial measurement session; however, blinding to appliance group could not be guaranteed.

### 2.5. Sample Size Considerations

Because of the retrospective design, the sample size was determined by the number of consecutive patients who fulfilled the predefined eligibility criteria and had complete records available during the study period; therefore, no formal a priori sample size calculation was performed before data collection. Of the 106 consecutively screened patients, 85 fulfilled the eligibility and data-completeness requirements and constituted the final study sample.

### 2.6. Statistical Analysis

Statistical analyses were performed using IBM SPSS Statistics for Windows, version 21.0 (IBM Corp., Armonk, NY, USA). Continuous variables were summarized as mean ± standard deviation (SD), and categorical variables were presented as frequencies and percentages. The distribution of continuous variables was assessed for normality before parametric analyses.

Baseline demographic and clinical characteristics were compared among the four treatment groups using one-way analysis of variance (ANOVA) for continuous variables and Pearson’s chi-square test for categorical variables. The expected cell frequencies were examined to verify the assumptions of the chi-square test; Fisher’s exact test was considered when these assumptions were not satisfied.

Within each treatment group, changes between pretreatment (T0) and post-treatment (T1) measurements were evaluated using paired-samples *t*-tests. For each outcome, the treatment-related change (Δ) was calculated as T1 − T0. Differences in Δ values among the four appliance groups were assessed using one-way ANOVA. When a significant overall group effect was identified, Tukey’s post hoc test was used for pairwise comparisons.

Effect sizes were calculated to complement null-hypothesis significance testing. For within-group paired comparisons between T0 and T1, standardized mean changes were expressed as Cohen’s dz. For intergroup comparisons of change scores (Δ), effect size was expressed as eta squared (η^2^). Where applicable, 95% confidence intervals were reported to provide information regarding the precision of the estimated effects. Effect sizes were interpreted together with their confidence intervals and *p*-values rather than statistical significance alone.

Because right and left maxillary first-molar crown inclination measurements were obtained from the same participant and were therefore not statistically independent, the mean of the right and left changes was used for the primary intergroup analysis of posterior crown inclination. Side-specific measurements were retained for descriptive purposes.

The principal outcomes of interest were changes in maxillary intercanine width, maxillary first-molar width, and projected palatal area. Maxillary first-molar clinical crown inclination was considered an exploratory outcome because it was derived from clinical crown morphology on STL models and does not represent root-level tooth inclination or true buccolingual torque. No formal multiplicity correction was applied to these secondary analyses. Therefore, *p*-values derived from the secondary and exploratory comparisons should be interpreted as descriptive and hypothesis-generating rather than confirmatory evidence. Particular caution was applied to isolated *p*-values near the conventional significance threshold, and interpretation emphasized the magnitude and overall pattern of the observed changes rather than statistical significance alone.

All tests were two-sided, and a *p*-value < 0.05 was considered statistically significant.

## 3. Results

A total of 85 adolescents were included in the final analysis, comprising 41 males and 44 females. The conventional MBT, Damon Q, Invisalign, and ClearCorrect groups included 22, 19, 21, and 23 participants, respectively. The mean chronological ages were 13.46 ± 1.83 years in the MBT group, 13.24 ± 1.32 years in the Damon Q group, 14.9 ± 1.88 years in the Invisalign group, and 14.03 ± 0.91 years in the ClearCorrect group. The corresponding mean treatment durations were 1.79 ± 0.41, 1.46 ± 0.24, 1.68 ± 0.33, and 1.54 ± 0.28 years, respectively. No statistically significant differences were detected among the four groups in chronological age, sex distribution, treatment duration, or ABO Discrepancy Index scores (all *p* > 0.05) (Table 1). The expected cell frequencies for the sex-distribution analysis satisfied the assumptions of Pearson’s chi-square test. Similarly, no statistically significant differences were detected among the groups for the evaluated pretreatment transverse dental measurements, projected palatal area, or maxillary first-molar crown inclination (*p* > 0.05) (Table 2).

Significant increases in maxillary transverse dimensions from T0 to T1 were observed within all four appliance groups (Table 3). Intergroup comparisons of change scores (Δ = T1 − T0) indicated differences in the magnitude of maxillary transverse changes among the treatment groups (Table 4). The greatest mean increase in maxillary intercanine width was observed in the Damon Q group (3.66 ± 1.34 mm), followed by ClearCorrect (3.12 ± 1.90 mm), Invisalign (2.06 ± 1.02 mm), and MBT (1.59 ± 0.71 mm).

For the maxillary premolar region, the mean increases in first-premolar width were 2.82 ± 1.59 mm, 4.79 ± 2.33 mm, 3.13 ± 2.09 mm, and 3.86 ± 2.14 mm for MBT, Damon Q, Invisalign, and ClearCorrect, respectively. Corresponding changes in second-premolar width were 3.07 ± 1.76 mm, 3.94 ± 2.12 mm, 2.98 ± 1.82 mm, and 3.48 ± 1.36 mm, respectively (Table 4).

In the maxillary molar region, first-molar width increased by 1.98 ± 1.12 mm in the MBT group, 3.69 ± 1.70 mm in the Damon Q group, 2.04 ± 1.04 mm in the Invisalign group, and 2.46 ± 1.42 mm in the ClearCorrect group. The corresponding increases in second-molar width were 0.63 ± 0.42 mm, 2.45 ± 1.19 mm, 1.11 ± 1.01 mm, and 1.44 ± 0.88 mm, respectively. Changes in transpalatal width also differed among the groups, with mean increases of 0.86 ± 0.24 mm for MBT, 2.32 ± 1.11 mm for Damon Q, 1.94 ± 1.07 mm for Invisalign, and 2.13 ± 1.30 mm for ClearCorrect (Table 4).

Projected palatal area increased significantly from T0 to T1 in all four groups (Table 3). The mean increases were 65.72 ± 16.47 mm^2^ for MBT, 115.16 ± 29.70 mm^2^ for Damon Q, 69.91 ± 20.48 mm^2^ for Invisalign, and 89.96 ± 21.77 mm^2^ for ClearCorrect, with a significant overall intergroup difference (*p* = 0.005) (Table 4).

Exploratory analysis of maxillary first-molar clinical crown inclination showed differences in the magnitude of change among the appliance groups. Side-specific descriptive analyses showed mean changes in UR16 crown inclination of 4.10 ± 2.14° for MBT, 2.90 ± 1.35° for Damon Q, 3.60 ± 1.16° for Invisalign, and 1.50 ± 0.33° for ClearCorrect. For UL26, the corresponding changes were 4.70 ± 2.31°, 3.10 ± 1.49°, 3.70 ± 1.22°, and 1.40 ± 0.47°, respectively (Table 4). Thus, the smallest side-specific changes in maxillary first-molar crown inclination were observed in the ClearCorrect group. These between-group differences represent comparative changes observed during the treatment period. Because no untreated control group was available and skeletal maturation was not directly assessed, the relative contributions of orthodontic treatment and physiological growth to these changes cannot be separated.

In the mandibular arch, the magnitude of transverse change was generally smaller than that observed in the maxilla (Table 3 and Table 4). Mean intercanine width increases were 1.29 ± 1.07 mm for MBT, 2.33 ± 1.30 mm for Damon Q, 1.43 ± 0.69 mm for Invisalign, and 1.57 ± 0.33 mm for ClearCorrect (*p* = 0.022). Nominal between-group differences (unadjusted *p* < 0.05) were observed for several mandibular transverse measurements; however, because these analyses were secondary and no formal multiplicity correction was applied, these findings should be regarded as exploratory and hypothesis-generating (Table 4).

## 4. Discussion

The present study compared dentoalveolar transverse changes observed during treatment in adolescents managed with two fixed treatment groups and two clear aligner systems. All four treatment groups demonstrated increases in maxillary transverse dimensions; however, the magnitude and distribution of these changes differed among groups. Greater mean increases at several maxillary transverse levels were observed in the Damon Q group, whereas comparatively smaller changes in maxillary first-molar crown inclination were observed in the ClearCorrect group. Projected palatal area increased in all groups, with the greatest mean increase observed in the Damon Q group. These between-group differences should be interpreted as comparative treatment-period observations rather than isolated appliance effects because physiological growth could not be quantified and an untreated control group was unavailable. Within these limitations, the null hypothesis of no between-group differences in the observed dentoalveolar changes was rejected [2,3].

The greater transverse changes observed with Damon Q at several maxillary levels are consistent with the ability of fixed treatment groups using broad preformed archwires to produce dentoalveolar arch development during alignment and leveling. In the present study, greater mean increases were observed in the Damon Q group than in the conventional MBT group at the intercanine, premolar, and molar levels. Nevertheless, these findings should not be interpreted as evidence that passive self-ligation itself is responsible for greater expansion. Previous investigations comparing self-ligating and conventional fixed appliances have reported inconsistent differences in transverse arch development, and randomized clinical evidence has not consistently demonstrated a clinically important advantage of self-ligating systems [1,4,7]. Differences in archwire form, treatment mechanics, baseline morphology, and individual biological response may contribute to the observed transverse changes. Therefore, the present findings describe appliance-associated patterns rather than establishing a specific biomechanical superiority of passive self-ligation. An additional consideration is that the two fixed appliance groups did not represent identical bracket–archwire systems. The conventional group used an MBT prescription with its corresponding sequential NiTi archwire protocol, whereas the passive self-ligating group used the Damon Q system with a CuNiTi archwire sequence. Although preformed archwires were used in both groups and archform selection was individualized according to pretreatment arch morphology and treatment objectives, differences in archwire form, dimensions, material properties, and bracket prescription may have influenced the transverse expression of treatment. In particular, differences in bracket prescription and bracket–archwire interaction may contribute to differences in posterior crown inclination and transverse arch development, while recent in vitro evidence indicates that archwire dimensions and geometry can influence load–deflection behavior and force-delivery characteristics [12]. Therefore, the greater transverse changes observed in the Damon Q group cannot be attributed to the self-ligating mechanism alone and should be interpreted as the response associated with the overall bracket–archwire system used in that group. Recent in vitro evidence has further demonstrated that the cross-sectional dimensions and edge geometry of CuNiTi orthodontic archwires can influence their load–deflection behavior and force-delivery characteristics under clinically relevant thermal conditions. Although these laboratory findings cannot directly explain the transverse changes observed in the present clinical sample, they highlight that archwire-related mechanical properties may contribute to differences in the biomechanical environment generated by fixed treatment groups [12].

Clear aligner treatment was also associated with increases in maxillary transverse dimensions. Previous studies have shown that transverse development with clear aligners is predominantly dentoalveolar and that the predictability of expansion varies according to tooth type and arch region [1,5]. Ma and Wang [9] reported significant increases in arch width with Invisalign, although predictability varied across tooth positions. Morales-Burruezo et al. [10] observed greater effectiveness in the premolar region than in some anterior and posterior regions, while Zhou et al. [11] reported lower predictability in the molar region than in the canine region. Similarly, Lione et al. [13] described regional differences in expansion predictability. These findings support the interpretation that transverse changes achieved with clear aligners should not be regarded as uniform bodily expansion across the dental arch. Importantly, the transverse increases observed with Invisalign and ClearCorrect in the present study should be interpreted as dentoalveolar arch changes and not as evidence of true skeletal maxillary expansion, which cannot be established from digital dental models alone.

An exploratory finding of the present study concerned differences in maxillary first-molar clinical crown inclination among the appliance groups. Comparatively smaller changes were observed in the ClearCorrect group, whereas larger changes were observed in the other groups, particularly MBT. This finding may reflect differences in the coronal expression of transverse tooth movement among the investigated treatment groups. However, the present methodology evaluated only the clinical crowns using STL surface models. Root position, movement of the center of resistance, buccolingual root inclination, and the relationship between the teeth and alveolar bone could not be determined. Accordingly, differences in clinical crown inclination should not be interpreted as evidence of superior torque control, bodily movement, or periodontal safety. Three-dimensional radiographic or other validated root-level assessments would be required to investigate these outcomes [14].

Projected palatal area increased in all four treatment groups, with the largest mean change observed in the Damon Q group. This pattern was generally consistent with the transverse linear measurements. However, projected palatal area should be interpreted cautiously because it represents a two-dimensional projection of a three-dimensional curved palatal surface rather than true palatal surface area or volume [15]. Although the registered models were evaluated using a standardized orientation and the same anatomically defined boundary-tracing procedure was applied across time points and treatment groups, the measurement remains dependent on consistent model orientation and manual boundary delineation. Therefore, projected palatal area should be regarded as a complementary morphological indicator of transverse arch change rather than a direct measure of skeletal or volumetric palatal expansion [16].

Changes in the mandibular arch were generally smaller than those observed in the maxilla. Significant intergroup differences were nevertheless identified for several mandibular transverse measurements. Damon Q showed the greatest mean increase in mandibular intercanine width, whereas differences at the premolar and molar levels were smaller. Previous studies evaluating self-ligating and conventional appliances have reported variable mandibular arch responses, and transverse changes during alignment may reflect a combination of archwire form, initial crowding, tooth inclination, and individual arch morphology [4,15]. Because the present study was not designed to isolate the contribution of each biomechanical component, the mandibular findings should primarily be interpreted as descriptive differences among the treatment groups.

From a clinical perspective, statistical differences among treatment groups do not necessarily indicate clinically meaningful superiority. Several of the intergroup differences in the present study were relatively small, and differences of approximately 0.5–1.0 mm in transverse width or 1–2° in crown inclination may have limited clinical relevance depending on the individual treatment objective. Furthermore, the study did not include a post-retention follow-up and therefore could not assess the long-term stability or relapse of the observed transverse changes. Accordingly, the T0–T1 increases represent changes observed during active orthodontic treatment and should not be interpreted as evidence of stable long-term arch expansion or growth modification. Periodontal outcomes, patient-reported outcomes, and the correspondence between digitally planned and clinically achieved tooth movements were also not evaluated. Consequently, appliance selection in adolescents should not be based solely on the magnitude of transverse change observed in this study. Individual malocclusion characteristics, periodontal boundaries, treatment objectives, patient preference, and the requirement for compliance should also be considered.

The age of the study population is particularly important when interpreting the present findings. Orthodontic treatment during adolescence occurs concurrently with physiological craniofacial and dentoalveolar development. Because skeletal maturation indicators such as cervical vertebral maturation stages were not consistently available in this retrospective sample, the contribution of normal growth to the observed transverse changes could not be quantified. Although no statistically significant differences in chronological age or pretreatment transverse measurements were detected among the treatment groups, this does not eliminate growth as a potential confounding factor. In addition, the absence of an untreated control group prevents separation of treatment-associated changes from physiological transverse development. The results should therefore be interpreted as comparative changes observed during treatment rather than as changes attributable exclusively to the treatment groups [17].

The retrospective and non-randomized design introduces additional limitations. Treatment modality was selected during routine clinical care according to clinical indication, patient preference, and appliance availability, creating the possibility of selection bias and residual confounding. Although chronological age, ABO Discrepancy Index, and pretreatment transverse measurements were evaluated to characterize baseline comparability among the groups, these variables cannot account for all baseline differences that may influence treatment response. In particular, initial crowding, arch-length discrepancy, buccal segment relationships, and patient-related factors potentially associated with compliance were not systematically balanced, matched, or included as covariates. Therefore, unmeasured differences among patients selected for fixed appliances versus clear aligners may have contributed to the observed between-group differences. Moreover, treatment protocols could not be standardized to the same extent as in a prospective randomized trial, and differences in archwire characteristics, aligner staging, refinements, or individual treatment mechanics may also have influenced the observed outcomes [17,18]. In addition, because the sample size was determined by the availability of eligible retrospective records rather than by a prospective a priori power calculation, the study may have had limited power to detect small between-group differences for some outcomes.

An additional methodological limitation concerns examiner blinding. All digital-model measurements were performed by a single calibrated examiner, and complete blinding to treatment modality could not be ensured because appliance-related information could potentially be identifiable from the available records or model characteristics. Although standardized measurement definitions and repeated-measurement reliability assessment were used to reduce measurement variability, intra-examiner reliability evaluates repeatability rather than eliminating potential observer-related measurement bias. This limitation may be particularly relevant to the exploratory crown-inclination assessment, which required construction of a best-fit clinical crown axis. Future studies should incorporate blinded assessment and, preferably, independent measurements by multiple calibrated examiners.

Compliance represents an additional consideration for the clear aligner groups. Patients were instructed to wear their aligners for at least 22 h/day, and compliance was evaluated clinically and through patient-reported wear time during follow-up. However, objective electronic wear-time monitoring was not available. Consequently, the actual contribution of compliance to individual treatment responses could not be quantified. This limitation is particularly relevant in an adolescent population, in whom removable appliance wear may vary between individuals.

Despite these limitations, the study has several methodological strengths. All patients were treated without extractions or auxiliary maxillary expansion appliances, pretreatment and post-treatment digital models were evaluated using standardized measurement definitions, and the analysis incorporated multiple aspects of dentoalveolar transverse change, including linear arch dimensions, projected palatal area, and posterior crown inclination. In addition, the four treatment groups were evaluated within the same study framework, allowing direct comparison of their observed transverse change patterns. Future prospective studies incorporating skeletal maturation assessment, objective aligner compliance monitoring, standardized treatment protocols, three-dimensional root-level evaluation, and long-term follow-up are needed to clarify the relative contribution of appliance mechanics and physiological growth to transverse changes during adolescent orthodontic treatment.

## 5. Conclusions

All four treatment groups demonstrated increases in dentoalveolar transverse dimensions during orthodontic treatment in this adolescent sample, although the magnitude and distribution of the observed changes differed among groups. Greater transverse increases at several maxillary levels were observed in the Damon Q group, whereas comparatively smaller changes in maxillary first-molar crown inclination were observed in the ClearCorrect group. Projected palatal area increased in all treatment groups, with the greatest mean increase observed in the Damon Q group.

These findings describe comparative dentoalveolar change patterns observed during treatment and should not be interpreted as evidence of overall superiority or isolated effects of any appliance system. Because skeletal maturation was not directly assessed, an untreated control group was unavailable, and root-level changes could not be evaluated, appliance-related effects cannot be separated from physiological growth and other potential treatment-related influences. Prospective controlled studies incorporating skeletal maturation assessment, standardized treatment protocols, and long-term post-retention follow-up are warranted to determine the stability of these dentoalveolar changes.

## Figures and Tables

**Table 1:** Comparison of the chronological ages, gender distributions, treatment time, expansion rate and ABO Discrepancy Index between the groups.

	Gender Distribution (Male/Female)	Chronological Age (Years) Mean ± SD	Treatment Time (Years) Mean ± SD	Expansion Rate (Transpalatal Width/Months)	ABO Discrepancy Index Mean ± SD
**Group I** **MBT Group** **(N = 22)**	10/12	13.46 ± 1.83	1.79 ± 0.41	0.04 ± 0.01 ^a^	12.15 ± 1.89
**Group II** **Damon Q Group** **(N = 19)**	9/10	13.24 ± 1.32	1.46 ± 0.24	0.13 ± 0.05 ^b^	13.49 ± 1.47
**Group III** **Invisalign Group** **(N = 21)**	11/10	14.9 ± 1.88	1.68 ± 0.33	0.09 ± 0.04 ^b^	12.8 ± 1.96
**Group IV** **ClearCorrect Group** **(N = 23)**	11/12	14.03 ± 0.91	1.54 ± 0.28	0.11 ± 0.02 ^b^	13.2 ± 2.01
***p***	0.878 *	0.442 †	0.903 †	0.044 †	0.165 †

SD: Standard deviation; N: Number; *: Results of Pearson chi-square test; †: Results of ANOVA test. Significantly different values are denoted by a different letter.

**Table 2:** Pre-treatment transversal measurements of the groups.

	MBT Mean ± SD	DAMON Q Mean ± SD	INVISALIGN Mean ± SD	CLEARCORRECT Mean ± SD	*p*
**MAXILLA**					
**Intercanine Width (3-3)**	31.02 ± 2.11	29.09 ± 2.5	30.1 ± 1.9	29.54 ± 1.7	NS
**Interfirst premolar Width (4-4)**	36.19 ± 1.47	34.17 ± 2.37	36.63 ± 2.4	35.04 ± 2.15	NS
**Intersecond premolar Width (5-5)**	38.5 ± 3.1	36.9 ± 1.82	37.35 ± 2.28	38.33 ± 2.6	NS
**Interfirst molar Width (6-6)**	44.76 ± 2.91	42.62 ± 3.32	44.63 ± 2.86	43.7 ± 2.39	NS
**Intersecond molar Width (7-7)**	44.91 ± 2.8	45.28 ± 2.6	45.79 ± 3.13	43.83 ± 1.4	NS
**Transpalatal width**	31.28 ± 3.2	32.27 ± 2.38	32.67 ± 2.1	29.98 ± 2.54	NS
**Palatal Surface (mm^2^)**	1053.72 ± 109.2	1190.58 ± 128.5	1069.42 ± 116.7	1035.53 ± 153.4	NS
**UR Molar Angulation (16)**	19.6 ± 2.86	20.5 ± 3.25	21.2 ± 2.43	19.8 ± 1.74	NS
**UL Molar Angulation (26)**	20.1 ± 3.6	21.8 ± 2.7	19.8 ± 2.9	20.3 ± 2.3	NS
**MANDIBLE**					
**Intercanine Width (3-3)**	24.4 ± 1.71	23.89 ± 2.62	24.27 ± 2.03	23.11 ± 2.11	NS
**Interfirst premolar Width (4-4)**	34.32 ± 1.62	33.58 ± 2.33	33.88 ± 2.42	33.91 ± 2.14	NS
**Intersecond premolar Width (5-5)**	39.73 ± 1.83	37.91 ± 2.27	38.53 ± 2.66	39.98 ± 2.9	NS
**Interfirst molar Width (6-6)**	41.72 ± 2.48	38.1 ± 1.98	40.95 ± 2.71	40.55 ± 3.08	NS
**Intersecond molar Width (7-7)**	49.15 ± 2.45	48.99 ± 2.16	52.33 ± 3.1	49.41 ± 2.7	NS

SD: Standard deviation; *p*: Results of One way—ANOVA test; NS: not-significant.

**Table 3:** Transversal changes in the treatment groups.

	MBT Mean ± SD		*p*	DAMON Q Mean ± SD		*p*	INVISALIGN Mean ± SD		*p*	CLEARCORRECT Mean ± SD		*p*
	T0	T1		T0	T1		T0	T1		T0	T1	
**MAXILLA**												
**Intercanine Width (3-3)**	31.02 ± 2.11	32.61 ± 2.66	***	29.09 ± 2.5	32.75 ± 2.69	***	30.1 ± 1.9	32.16 ± 2.4	***	29.54 ± 1.7	32.66 ± 2.47	***
**Interfirst premolar Width (4-4)**	36.19 ± 1.47	39.01 ± 2.03	***	34.17 ± 2.37	38.96 ± 3.05	***	36.63 ± 2.4	39.76 ± 3.9	***	35.04 ± 2.15	38.9 ± 2.66	***
**Intersecond premolar Width (5-5)**	38.5 ± 3.1	41.57 ± 2.81	***	36.9 ± 1.82	40.84 ± 2.57	***	37.35 ± 2.28	40.33 ± 3.74	***	38.33 ± 2.6	41.81 ±3.08	***
**Interfirst molar Width (6-6)**	44.76 ± 2.91	46.74 ± 3.07	***	42.62 ± 3.32	46.31 ± 2.88	***	44.63 ± 2.86	46.67 ± 3.69	***	43.7 ± 2.39	46.16 ± 3.2	***
**Intersecond molar Width (7-7)**	44.91 ± 2.8	45.54 ± 4.11	***	45.28 ± 2.6	47.73 ± 3.2	***	45.79 ± 3.13	46.9 ± 3.17	***	43.83 ± 1.4	45.27 ± 3.07	***
**Transpalatal width**	31.28 ± 3.2	32.14 ± 2.89	***	32.27 ± 2.38	34.59 ± 2.75	***	32.67 ± 2.1	34.61 ± 2.96	***	29.98 ± 2.54	32.11 ± 2.87	***
**Palatal Surface (mm^2^)**	1053.72 ± 109	1119.44 ± 99	***	1190.58 ± 128	1305.74 ± 117	***	1069.42 ± 116	1139.33 ± 121	***	1035.53 ± 153	1125.49 ± 137	***
**UR Molar Angulation (16)**	19.6 ± 2.86	23.7 ± 1.79	***	20.5 ± 3.25	23.4 ± 3.11	***	21.2 ± 2.43	24.8 ± 2.6	***	19.8 ± 1.74	21.3 ± 1.86	***
**UL Molar Angulation (26)**	20.1 ± 3.6	24.8 ± 2.91	***	21.8 ± 2.7	24.9 ± 2.44	***	19.8 ± 2.9	23.5 ± 1.98	***	20.3 ± 2.3	21.7 ± 2.14	***
**MANDIBLE**												
**Intercanine Width (3-3)**	24.4 ± 1.71	25.69 ± 1.88	***	23.89 ± 2.62	26.22 ± 2.83	***	24.27 ± 2.03	25.7 ± 2.11	**	23.11 ± 2.11	24.68 ± 2.13	**
**Interfirst premolar Width (4-4)**	34.32 ± 1.62	35.04 ± 2.02	**	33.58 ± 2.33	35.31 ± 3.31	***	33.88 ± 2.42	34.58 ± 3.06	**	33.91 ± 2.14	34.72 ± 2.71	**
**Intersecond premolar Width (5-5)**	39.73 ± 1.83	39.95 ± 1.69	NS	37.91 ± 2.27	38.86 ± 2.16	***	38.53 ± 2.66	39.72 ± 2.94	**	39.98 ± 2.9	40.51 ± 3.18	**
**Interfirst molar Width (6-6)**	41.72 ± 2.48	42.25 ± 2.77	**	38.1 ± 1.98	39.13 ± 2.49	***	40.95 ± 2.71	41.67 ± 3.7	*	40.55 ± 3.08	41.56 ± 3.27	*
**Intersecond molar Width (7-7)**	49.15 ± 2.45	49.31 ± 3.06	NS	48.99 ± 2.16	49.68 ± 3.22	**	52.33 ± 3.1	52.79 ± 3.51	NS	49.41 ± 2.7	49.7 ± 3.66	NS

SD: Standard deviation; *p*: Results of Paired *t*-test; NS: not-significant (*p* > 0.05); * *p* < 0.05, ** *p* < 0.01, *** *p* < 0.001.

**Table 4:** Statistical comparison of differences between groups.

	MBT Mean ± SD	DAMON Q Mean ± SD	INVISALIGN Mean ± SD	CLEARCORRECT Mean ± SD	*p*	*Post-Hoc Tests*					
						1-2	1-3	1-4	2-3	2-4	3-4
**MAXILLA**											
**Intercanine Width (3-3)**	1.59 ± 0.71	3.66 ± 1.34	2.06 ± 1.02	3.12 ± 1.9	0.000	***	**	***	**	NS	**
**Interfirst premolar Width (4-4)**	2.82 ± 1.59	4.79 ± 2.33	3.13 ± 2.09	3.86 ± 2.14	0.005	***	**	***	**	*	NS
**Intersecond premolar Width (5-5)**	3.07 ± 1.76	3.94 ± 2.12	2.98 ± 1.82	3.48 ± 1.36	0.017	*	NS	NS	**	NS	**
**Interfirst molar Width (6-6)**	1.98 ± 1.12	3.69 ± 1.7	2.04 ± 1.04	2.46 ± 1.42	0.011	***	NS	**	**	**	**
**Intersecond molar Width (7-7)**	0.63 ± 0.42	2.45 ± 1.19	1.11 ± 1.01	1.44 ± 0.88	0.003	***	*	**	**	**	*
**Transpalatal width**	0.86 ± 0.24	2.32 ± 1.11	1.94 ± 1.07	2.13 ± 1.3	0.014	***	**	***	*	NS	NS
**Palatal Surface (mm^2^)**	65.72 ± 16.47	115.16 ± 29.7	69.91 ± 20.48	89.96 ± 21.77	0.005	***	NS	*	**	**	*
**UR Molar Angulation (16)**	4.1 ± 2.14	2.9 ± 1.35	3.6 ± 1.16	1.5 ± 0.33	0.000	**	NS	***	NS	***	***
**UL Molar Angulation (26)**	4.7 ± 2.31	3.1 ± 1.49	3.7 ± 1.22	1.4 ± 0.47	0.000	**	NS	***	NS	***	***
**MANDIBLE**											
**Intercanine Width (3-3)**	1.29 ± 1.07	2.33 ± 1.3	1.43 ± 0.69	1.57 ± 0.33	0.022	*	NS	NS	*	**	NS
**Interfirst premolar Width (4-4)**	0.72 ± 0.33	1.73 ± 0.98	0.7 ± 0.07	0.81 ± 0.4	0.016	***	NS	NS	***	**	NS
**Intersecond premolar Width (5-5)**	0.22 ± 0.04	0.95 ± 0.41	1.19 ± 0.61	0.53 ± 0.14	0.003	***	***	**	NS	*	**
**Interfirst molar Width (6-6)**	0.53 ± 0.17	1.03 ± 0.37	0.72 ± 0.23	1.01 ± 0.36	0.019	**	*	**	NS	NS	NS
**Intersecond molar Width (7-7)**	0.16 ± 0.05	0.69 ± 0.24	0.46 ± 0.71	0.29 ± 0.11	0.033	***	*	NS	NS	*	NS

SD: Standard deviation; *p*: Results of One way—ANOVA test; NS: not-significant (*p* > 0.05); * *p* < 0.05, ** *p* < 0.01, *** *p* < 0.001.

## Data Availability

The data presented in this study are available on reasonable request from the corresponding author.

## References

[1] Galluccio G., De Stefano A.A., Horodynski M., Impellizzeri A., Guarnieri R., Barbato E., Di Carlo S., De Angelis F. (2023). Efficacy and Accuracy of Maxillary Arch Expansion with Clear Aligner Treatment. Int. J. Environ. Res. Public Health.

[2] Lombardo E.C., Fanelli S., Pavoni C., Cozza P., Lione R. (2023). Maxillary Response Induced by Rapid Palatal Expansion vs. Clear Aligners: A Short-Term Retrospective Evaluation of the Dento-Alveolar Effects in Mixed Dentition. Appl. Sci..

[3] Kalekar A.A., Manchanda J., Chavan S., Bhad W.A., Atram H., Badu P., Tarde P. (2024). Effectiveness of maxillary arch expansion using clear aligners in adult patients: A systematic review and meta-analysis. Australas. Orthod. J..

[4] Kaşıkçıoğlu T., Pamukçu H., Arman Özçırpıcı A., Polat Özsoy Ö. (2023). Comparison of the initial levelling efficiency of two different self-ligating brackets. Acta Odontol. Turc..

[5] Vidal-Bernárdez M., Vilches-Arenas Á., Sonnemberg B., Solano-Reina E., Solano-Mendoza B. (2021). Efficacy and predictability of maxillary and mandibular expansion with the Invisalign^®^ system. J. Clin. Exp. Dent..

[6] Cangialosi T.J., Riolo M.L., Owens S., Dykhouse V.J., Moffitt A.H., Grubb J.E., Greco P.M., English J.D., James R. (2004). The ABO discrepancy index: A measure of case complexity. Am. J. Orthod. Dentofac. Orthop..

[7] Inchingolo A.M., Ceci S., Coloccia G., Azzollini D., Malcangi G., Mancini A., Inchingolo F., Trerotoli P., Dipalma G., Patano A. (2023). Predictability and Effectiveness of Nuvola^®^ Aligners in Dentoalveolar Transverse Changes: A Retrospective Study. Biomedicines.

[8] Loberto S., Pavoni C., Fanelli S., Lugli L., Cozza P., Lione R. (2024). Predictability of Expansion Movements Performed by Clear Aligners in Mixed Dentition in Both Arches: A Retrospective Study on Digital Casts. BMC Oral Health.

[9] Ma S., Wang Y. (2023). Clinical outcomes of arch expansion with Invisalign: A systematic review. BMC Oral Health.

[10] Morales-Burruezo A., Gandia J.L., Cibrian R., Paredes V. (2020). Arch expansion with the Invisalign system: Efficacy and predictability. Am. J. Orthod. Dentofac. Orthop..

[11] Zhou Y., Mao J., Xu T., Hu H. (2020). Efficiency of upper arch expansion with the Invisalign system. Am. J. Orthod. Dentofac. Orthop..

[12] Basilicata M., Bruno G., Ponticelli G.S., Bollero P., Docimo R., Fava A., Guarino S., Montanari R., Palombi A., Scuderi A. (2026). Thermo-Mechanical Characterization of Innovative Side-Rounded Superelastic CuNiTi Orthodontic Wires. J. Funct. Biomater..

[13] Lione R., Paoloni V., Franchi L., Cozza P. (2021). Maxillary arch development with the Invisalign system: Efficacy and predictability. Am. J. Orthod. Dentofac. Orthop..

[14] Zhao X., Xu T., Shu G. (2017). Maxillary expansion efficiency with clear aligner and its possible influencing factors. Am. J. Orthod. Dentofac. Orthop..

[15] Pandis N., Polychronopoulou A., Eliades T. (2007). Self-ligating vs conventional brackets in the treatment of mandibular crowding: A prospective clinical trial of treatment duration and dental effects. Am. J. Orthod. Dentofac. Orthop..

[16] Fleming P.S., Lee R.T., Marinho V., Johal A. (2013). Comparison of maxillary arch dimensional changes with passive and active self-ligation and conventional brackets in the permanent dentition: A multicenter, randomized controlled trial. Am. J. Orthod. Dentofac. Orthop..

[17] Franchi L., Baccetti T., Camporesi M., Lupoli M. (2006). Maxillary arch changes during leveling and aligning with fixed appliances and low-friction ligatures. Am. J. Orthod. Dentofac. Orthop..

[18] Chen J., Wen J., Huang L., Zhang L., Han L., Li H. (2022). Comparisons of maxillary incisor retraction effects for patients with first premolar extractions between Damon Q and Invisalign^®^: A retrospective study. Medicine.

